# Experiences and preferences of people with stroke and caregivers, around supports provided at the transition from hospital to home: a qualitative descriptive study

**DOI:** 10.1186/s12883-024-03767-0

**Published:** 2024-07-22

**Authors:** Geraldine O’Callaghan, Martin Fahy, Sigrid O’Meara, Sebastian Lindblom, Lena von Koch, Peter Langhorne, Rose Galvin, Frances Horgan

**Affiliations:** 1 iPASTAR Collaborative Doctoral Award Programme, School of Physiotherapy, RCSI University of Medicine and Health Sciences, 123 St. Stephen’s Green, Dublin, D02 YN77 Ireland; 2 iPASTAR Collaborative Doctoral Award Programme, RCSI School of Population Health Sciences, RCSI University of Medicine and Health Sciences, 123 St. Stephen’s Green, Dublin, D02 YN77 Ireland; 3 Department of Neurobiology, Care Sciences and Society, Karolinska Institutet, Alfred Nobels allé 23, Huddinge, Stockholm, Sweden; 4 Theme of Heart & Vascular and Neuro, Karolinska University Hospital, Stockholm, 14186 Sweden; 5 School of Cardiovascular and Metabolic Health (SCMH), University of Glasgow, 126 University Place, Glasgow, GT12 8TA Scotland; 6 School of Allied Health, Faculty of Education and Health Sciences, Ageing Research Centre, Health Research Institute, University of Limerick, Limerick, V94 T9PX Ireland

**Keywords:** MESH terms: stroke, Patient discharge, Patient experience, Patient perspective, Patient care planning, Recovery of function, Rehabilitation, Qualitative research

## Abstract

**Background:**

Transitioning home from the structured hospital setting poses challenges for people with stroke (PWS) and their caregivers (CGs), as they navigate through complex uncertainties. There are gaps in our understanding of appropriate support interventions for managing the transition home. In this qualitative study, we explored the perspectives of PWS and their CGs regarding their support experiences and preferences during this period.

**Methods:**

Between November 2022 and March 2023, and within six months of hospital discharge, audio-recorded, semi-structured interviews were conducted with PWS and CGs. All interviews were transcribed, imported into NVivo software, and analysed using reflexive thematic analysis.

**Results:**

Sixteen interviews were conducted, nine with PWS and seven with CGs. Four themes relevant to their collective experiences and preferences were identified: (i) Need for tailored information-sharing, at the right time, and in the right setting; (ii) The importance of emotional support; (iii) Left in limbo, (iv) Inequity of access. Experiences depict issues such as insufficient information-sharing, communication gaps, and fragmented and inequitable care; while a multi-faceted approach is desired to ease anxiety and uncertainty, minimise delays, and optimise recovery and participation during transition.

**Conclusions:**

Our findings highlight that regardless of the discharge route, and even with formal support systems in place, PWS and families encounter challenges during the transition period. The experiences of support at this transition and the preferences of PWS and CGs during this important period highlights the need for better care co-ordination, early and ongoing emotional support, and equitable access to tailored services and support. Experiences are likely to be improved by implementing a partnership approach with improved collaboration, including joint goal-setting, between PWS, CGs, healthcare professionals and support organisations.

**Supplementary Information:**

The online version contains supplementary material available at 10.1186/s12883-024-03767-0.

## Background

Stroke a significant public health concern worldwide, and although age-standardised rates of incidence, prevalence, and mortality from stroke are generally decreasing in Western countries, rates among young adults are increasing, and there is a notable increase in the overall burden [1]. While advances have been made in acute stroke care, the journey continues following hospital discharge, and for many people with stroke (PWS), and their caregivers (CGs), moving from the structured hospital environment back to the community can be a daunting experience, as they navigate complex challenges and uncertainties.

Typically, when individuals are discharged from the acute hospital or in-patient rehabilitation setting either home directly; to early supported discharge (ESD) teams; or to community services, there is a ‘care transition’ between settings. A ‘care transition’ is defined by the American Geriatric Society as “a set of actions designed to ensure the co-ordination and continuity of health care as patients transfer between different locations or different levels of care” [2].

The transition to home period refers to the six months following a discharge from hospital, where PWS have not yet entered the chronic phase [3, 4]. During this phase, a range of healthcare services and supports are provided to facilitate a smooth transition from hospital-to-community. Caregivers, serving as crucial support networks, play a vital role in this phase, significantly influencing treatment adherence and overall recovery [5]. However, a significant number of PWS and their families describe this period as complex [5]. Limited communication, information-sharing and coordination of care have been identified as significant obstacles to a seamless transition. Individuals often experience challenges accessing services and describe a fragmented healthcare system, where PWS and their families experience less than optimal progress in their post-acute stroke recovery [5–7]. They emphasise a number of unmet needs during this time [5, 8, 9], where the delivery of interventions at transition are not aligned with their needs [10], and where those with the more significant needs are often disproportionally affected [11]. Even among those who transition home via ESD, where tailored rehabilitation is delivered at home facilitating earlier discharge [12], they experience ongoing challenges reintegrating to life after stroke [13]. With an increased focus on person-centred, integrated healthcare that provides timely and equitable access to quality care for all [6, 14], healthcare providers have a responsibility to provide services at transition that ensure continuity of care and optimise outcomes for PWS and CGs. However, the challenges healthcare providers have in providing comprehensive care at these transitions can lead to many unmet needs for PWS, encompassing social and clinical care and facilitating participation in activities like driving, work, and leisure, while ensuring access to services, information, and support [15], and for CGs, including support in caring for their loved one and caring for themselves [16]. Furthermore, our understanding of effective support interventions to manage the transition to home after a stroke remains incomplete [17]. Healthcare providers must understand the support perspectives and preferences of PWS and CGs to deliver effective support services. However, there remains a lack of understanding about the perspectives and preferences of PWS and CGs regarding the most suitable support types for their unique needs during the transition process. Addressing these knowledge gaps is essential to developing person-centred, targeted strategies that can improve PWS and their carers’ overall post-stroke outcomes and experiences.

## Methods

### Aim

The aim of this study was to explore the experiences of people with stroke and caregivers around the supports they received during the transition to home, as well as their preferences for supports to be received at this time.

### Design

A qualitative descriptive approach using semi-structured interview methodology and reflexive thematic analysis [18], was used to explore the experiences and preferences of PWS and their families/informal CGs, around supports delivered at transition to home. The study is presented in accordance with the checklist provided by the Consolidated Criteria for Reporting Qualitative Research (COREQ) [19] (Supplemental data S1). Interview scripts for PWS and CGs can be found in Supplemental data S2. Interviews were carried out in-person or via telecommunication platform (Microsoft Teams).

### Setting

The Irish health system comprises both public and private sectors, funded through taxation and health insurance. Generally all PWS can access free public health services. However, some services and supports such as GP visits, prescribed medications, and aids and appliances, may only be free of charge for those eligible for a state funded Medical Card. Eligibility for a medical card is based on income thresholds, or other criteria which may be difficult to navigate. Initially, stroke care in Ireland adheres to a structured pathway, beginning with emergency response and acute stroke treatment, and most patients, but not all, access a recommended stroke unit for post-acute care [20]. However, the concept of organised stroke care diminishes when many patients encounter challenges accessing consistent post-acute support [20], an aspect currently not tracked in the national stroke audit [21]. Rehabilitation services, provided publicly through inpatient hospitals and community-based centres, are essential for the long-term recovery of PWS. Similarly, support services such as home care assistance play a crucial role. However, variations exist depending on factors such as geographic location, availability or access to home supports, and the types of rehabilitation services provided, including inpatient rehabilitation, ESD, specialised community rehabilitation, and generic primary care [22]. Many individuals face extended waiting periods in hospital or at home for access to ongoing rehabilitation or home support. The availability and accessibility of follow-up care and secondary prevention measures are inconsistent and fragmented, with data on much best practice care [6, 7], such as ongoing needs assessments, personalised rehabilitation and transition planning, and secondary stroke prevention, not consistently being tracked in Ireland [23].

### Sampling and recruitment

This study was conducted alongside an observational cohort study that profiled the outcomes and documented the needs of PWS during the period of their transition from structured services to home [24]. PWS were recruited across three Irish hospital sites, including two acute urban-based Model 4 hospitals (Site 1 & 2) and a Model 3 regional hospital (Site 3). In Ireland, Model 4 hospitals typically offer more comprehensive specialised services to larger populations, while Model 3 hospitals deliver broader less specialised services to local communities. Individuals were included provided they had a confirmed stroke diagnoses, and were aged 18 or above, living at home post-discharge, capable of giving informed consent, and possessed sufficient verbal communication to participate in an interview.

In this qualitative study PWS were interviewed separately from the observational study data collection. We adopted a pragmatic approach and applied a maximum variation sampling strategy to PWS and CGs. A maximum variation sample is constructed by identifying key dimensions of variations and then finding cases that vary from each other as much as possible [25].

#### People with stroke (PWS)

We recruited PWS discharged through pathways of home direct, following in-patient rehabilitation, and via ESD. The key dimensions of variations used to increase heterogeneity between participants were the discharge pathway, and individuals who had either a high (7–9) or low (1–3) number of unmet needs. Level of need was established at the initial data collection point of the cohort study using a self-reported needs survey designed by McKevitt et al. [8] Secondary factors included gender, over and under 65, and geographical regions, contributing to additional variability in the sample.

#### Caregivers (CGs)

We included spouses or family members who supported their loved ones’ participation in the cohort study, or were identified by PWS as providing care and assistance to them. They represented different discharge pathways and geographical regions.

Invitation letters and participant information were provided to all participants. Following a minimum one-week consideration period, interested participants were contacted by the lead researcher (GOC) to discuss and obtain informed consent. GOC then arranged interviews.

We aimed to ensure a meaningful sample size for semi-structured interviews of between 5 and 50 [26, 27], with a clear focus on data richness rather than saturation [28, 29]. Considering maximum variation sampling, a total of 17 participants were proposed (10 PWS, 7 CGs) to be reviewed in the field. A maximum variation sample table for PWS and CGs can be found in Table 1.

**Table 1:** Maximum variation sample plan for PWS and CGs

PWS*	Severity of need		Total	CGs	Geographic Location			Total
	High	Low			Site 1	Site 2	Site 3	
Home direct	2	2	4	Supporting home direct	1	1	1	3
Home after inpatient rehabilitation	1	1	2	Supporting after inpatient rehabilitation	1		1	2
Home via ESD	2	2	4	Supporting Home via ESD	1	1		2
Total	5	4	10	Total	3	2	2	7

### Data collection

Semi-structured interviews were conducted between November 2022 and March 2023. This was a period when COVID community restrictions had eased but caution persisted in hospital settings. Interviews were facilitated via home visits, telephone, or telecommunication platforms like Microsoft Teams, depending on the participants’ preferences. Most individuals were interviewed in their homes which enabled open and candid communication. Some interviews were conducted in dyads to address participant preferences. The intention was to conduct interviews between four and six months of hospital discharge, which, while still considered a transition period, would allow PWS and CGs an opportunity to have reflected on their transition to home experience, recovery to date and future needs. Three participants opted for earlier interviews. The interview duration ranged between 24 min and 77 min.

The interview guides were developed based on insights from our previous systematic review [17], patient and public involvement (PPI) collaborations, relevant literature around experiences, challenges and opportunities at hospital-to-home transitions after stroke, and the Template for Intervention Description and Replication (TIDieR) checklist [30]. An interview guide can be found in Supplemental data S2.

All interviews were carried out by the primary researcher (GOC), a female physiotherapist working in stroke care and a doctoral student. As part of her postgraduate education, GOC received training in qualitative research, focusing on equipping early-career researchers with skills in qualitative research methods. Interviews were audio-recorded, transcribed verbatim by a third-party transcriber, and prior to analysis were returned to participants for member checking [31].

### Data analysis

The transcripts were uploaded to NVivo 12 software [32] and thematically analysed using Braun and Clarke’s 6 stage model of ‘reflexive’ thematic analysis (RTA): (1) data familiarisation and writing familiarisation notes; (2) systematic data coding; (3) generating initial themes from coded and collated data; (4) developing and reviewing themes; (5) refining, defining and naming themes; and (6) writing the report [18, 33]. RTA was selected because of its flexibility in terms of theoretical independence (without being a-theoretical) and ability to choose how to enact the analysis; its emphasis is on the researcher’s reflexivity, and an ability to maintain a focus on the overarching research questions [18, 34]. Framed in a constructionist epistemology, inductive analysis involved an open, flexible and iterative process of reading and re-reading transcripts, summarising each transcript, and following with line by line coding, leading to clustering and theme generation. The data was read, re-read, and coded by GOC. Reflexive practice was supported by field notes, and a reflexive diary was completed at the end of each interview. Reflexive practice was also facilitated through peer debriefing between GOC and her supervisors (FH and RG) and co-authors (MF, SO’M, LVK, SL, PL). Themes (patterns of shared meaning) were generated at semantic and latent levels. Overarching themes and concepts generated through this collaborative and reflexive process were related back to the research questions.

### Patient and public involvement (PPI)

PPI stroke champions, five individuals who had experienced a stroke and one caregiver, contributed to the methodological development of this study; the ethics submission process; study recruitment; and the development and testing of the interview guides, after which minor adjustments were made to enhance question clarity. Further collaboration involved presenting the results to PPI stroke champions, inviting open discussion and space for their perspectives, interpretations, and reflections to be shared. Insights informed the discussion section, highlighting implications for practice, policy, and future research.

### Ethics approval

Ethics approval was obtained from Ethics (Medical Research) Committee - Beaumont Hospital (ref: 22/41), from Clinical Research Ethics Committee, Galway University Hospital (ref: C.A. 2872); and from Reference Research Ethics Committee Midlands Area and Corporate (Regional Health Area B) (ref: RRECB1022FH).

## Results

A total of 16 interviews were conducted, with 9 PWS and 7 CGs. A summary of participant demographics can be found in Table 2 (PWS) and Table 3 (CGs). Among the PWS, age ranged between 47 and 79, four of whom were > 65, while 6 were male and 4 female. Among the 7 CGs, 4 were spouses of the PWS, and 3 were children. Three PWS were interviewed as part of a dyad with their spouses. Despite utilising maximum variation sampling to identify individuals with distinctly high or low needs, recruitment challenges were encountered as many participants fell within the moderate range of unmet needs (6–9), and in Site 1 we were unable to recruit a PWS with low need. Further detail of the maximum variation sampling can be found in Supplemental data S3.

**Table 2:** Participant demographics: people with stroke (PWS) (*n* = 9)

ID	Age	Gender	Length of stay (LOS)*	Time discharge to interview**	Discharge Pathway	Level of need	Site	Interviewed individually or as part of dyad
PWS_1	70	Male	7 days	42 days	ESD	Moderate	Site 1	Individually
PWS_2	54	Male	81 days	99 days	Home direct	Moderate	Site 3	Dyad
PWS_3	55	Male	265 days	19 days	Rehab	High	Site 3	Dyad
PWS_4	74	Female	11 days	116 days	Home direct	Low	Site 3	Individually
PWS_5	66	Male	4 days	135 days	ESD	Moderate	Site 2	Individually
PWS_6	55	Female	252 days	83 days	Rehab	High	Site 3	Individually
PWS_7	79	Male	17 days	103 days	ESD	Moderate	Site 2	Individually
PWS_8	56	Male	39 days	105 days	Rehab to ESD	Low	Site 2	Dyad
PWS_9	47	Female	12 days	175 days	ESD	Low	Site 2	Individually

*Median LOS was 17 days (range 4-265)

**Median time from discharge to interview was 103 days (range 42–175)

**Table 3:** Participant demographics: caregivers (CGs) (*n* = 7)

ID	Gender	Relationship to PWS	Employment status	Discharge Pathway Supporting	Site	Interviewed individually or as part of dyad
CG_1	Male	Son	Working full time	Rehab and ESD	Site 1	Individually
CG_2	Female	Wife	Working full time	ESD and Rehab	Site 2	Individually
CG_3	Female	Wife	Working full time	Home direct	Site 3	Dyad
CG_4	Female	Wife	Working full time	Home direct	Site 3	Dyad
CG_5	Female	Daughter	Working full time	Home direct	Site 2	Individually
CG_6	Female	Wife	On carer leave	Rehab and ESD	Site 1	Dyad
CG_7	Female	Daughter	Working full time	ESD	Site 1	Individually

Our findings are described through four key themes, namely (i) Need for tailored information-sharing, at the right time, and in the right setting; (ii) The importance of emotional support; (iii) Left in limbo, (iv) Inequity of access.

I. Need for tailored information-sharing, at the right time, and in the right setting.

This theme captures the experiences of information provided as PWS and CGs navigated the transition to home. It also reflects the specific preferences of individuals regarding the information they require during this period, derived from positive experiences or articulated needs for specific resources.

In general, PWS felt frustrated by the lack of specific details about their stroke, its implications and ongoing management, plans for and life following transition to home; while for CGs information was provided at stressful times, or in ways that were difficult to comprehend, especially due to COVID-19 restrictions.

“None of this was, not that it wasn’t properly explained, it was explained to you at a very stressful time that you can’t absorb anything and obviously a time when you couldn’t bring a family member with you because we had the whole Covid going on, you know….So there was information, I think, missed, probably not their fault, not mine either but there was definitely information missing or it was never properly explained” (CG02).

A lack of dedicated time or structured sessions for information delivery was a common issue

“well no, there really wasn’t, nearly every day the doctor would come in but he would just ask you your name and how you felt that day and so on and how you were getting on. But nobody actually sat down and said, you have had a stroke and you can expect this type of thing to happen. We don’t know how bad it was, we are trying to figure that out. There was none of that” (PWS04).

At times, PWS felt that assumptions were made about the level of information needed by them, which shaped subsequent conversations. There was also a perceived reluctance for healthcare professionals (HCPs) to have some of the more difficult conversations

“only the OT said to me one of the days before I came home I hate having these conversations when no one else has had them but there is a likelihood that you will always have it, you might never drive again” (PWS06).

For those with positive experiences with the information they received about their stroke, ongoing recovery, and what services and supports to expect at transition to home, they felt being ready to ask questions and actively seeking out individualised information was key.

PWS who transitioned home to ESD discussed their experience of having ready access to their own health information and building trust with their healthcare team.

“one of the most important things they did was, when they explained to me, they had a folder there that was left here for the duration of the eight or ten weeks and when we would have our session which could last for an hour, an hour and a half, for the last five minutes or ten minutes they would sit down and do up the report, and that was there for me to read…” (PWS01).

PWS and CG emphasised their preference for improved communication and tailored information-sharing designed to meet the unique needs of each individual,

“For me verbal was fine but for other people written would be better, but written for them not written as in a leaflet that goes out generally to everyone. I mean I had a bled and to tell you the truth that is the only type of stroke I am interested in” (PWS06).

delivered in their preferred format,

“Paper format is good, a beautifully written book… but personally I would be googling everything so I would have been a big reader of the stroke.org.uk, the UK stroke entity.…various methods and multiple timings for providing information is extremely important, and I would say that actually rather than quickly just moving on, giving the space to the patient is critical” (PWS05).

at the right time, and in the right setting

“For him (ongoing information and support) would have been better coming up nearer to Christmas because he had kind of digested everything that had happened… And I think if you can provide it in the home rather than him having to get up and get out and get washed and go somewhere again because that adds to the stress of it all, especially in the first six months. The more times you have to get up and get ready, drive somewhere, park, you know, look for a parking space, walk into the appointment, wait in the waiting room. That all adds to stress I think for the whole family because as I say he is relying on us” (CG05).

II. The importance of emotional support.

This theme delves into the emotional support received by PWS and CGs as they transitioned home and encompasses their unique preferences for emotional support along the transition journey.

PWS and CGs experienced a range of emotions across the transition, from nervousness, worry and uncertainty, to happiness and relief. PWS often felt conflicted about their readiness and abilities at transition, while CGs struggled with the sudden responsibility of caregiving, feeling overwhelmed and doubting their ability to cope. For all, the impact of stroke was profound, affecting family dynamics and everyone’s emotional well-being, including children. Both PWS and CGs felt overlooked in terms of emotional support, that their emotional health wasn’t given enough attention. Some PWS perceived their post-stroke emotions being invalidated or dismissed, as they were told to

“Just go home and forget you ever got it” (PWS04).

Meanwhile, CGs felt whatever emotional support was available was targeted towards the PWS. Their experience of receiving emotional support was one where the GP recommended medication, or counselling was advised but not provided. Sourcing counselling independently and/or paying for it themselves created a barrier for them.

“What they should have is someone who comes in, that’s in-house, a counsellor here and they will be in contact with you and your family if you require that service but we are here to offer this. But that is not available, as far as I am aware it is not available because it was never mentioned to me. You had to source it basically yourself or go to your GP and get medicated” (CG02).

PWS acknowledged the significance of emotional support from family, friends, and colleagues however, they recognised a need to talk to someone who was not their loved one about emotional challenges. Those who transitioned home to ESD felt they were offered good emotional support from the team,

“Once or twice I was a bit down and they detected it and we talked… after that then I was fine” (PWS01).

However, they experienced a gap in services where there were mental health concerns that might benefit from the expertise of trained professionals.

The positive experiences of emotional support derived from stroke support organisations or through connections they made with others undergoing similar experiences was emphasised by both PWS and CGs. Peer support derived from fellow PWS met during their hospital stay provided both practical and emotional support;

“I just sort of talking to people, when I was in the hospital I was talking to a couple of the lads and they were sort of saying that the most important thing for them was to know exactly what the problems that they would encounter was there for them to overcome” (PWS07).

However, this support was only of temporary benefit as once discharged as “they were now on their own journey” (PWS07), preferring more tailored emotional support from that point.

CGs described the need for in-person peer support facilitated early in the recovery process, and to be ongoing beyond the initial phase.

“but it would be nice to link up with people who are at the same stage of the process and then also (something) for people who are further down the process… I don’t know how they would do it. Once again if there was some kind of group, you know, if there was that peer to peer kind of a face to face meeting that you could go along” (CG03).

III. Left in limbo.

This theme encapsulates the feelings of uncertainty and being stuck in an in-between state, as PWS and CGs encountered fragmented care coordination, faced unfulfilled expectations regarding continuity of care, and endured prolonged waits for services and supports. Specific preferences of PWS and CGs are less defined, however could be described as an overarching desire for integrated care at transition.

The collective experiences of PWS and CGs reveal a lack of care coordination throughout the transition to home trajectory. Disjointed discharge planning led to fragmented decision-making. PWS and CGs were excluded from decisions about discharge and ongoing care, and many experienced sudden discharges, leading to feelings of confusion and a sense of being unprepared.

“I got the sense that there was no discharge planned and that is why I was in hospital and the discharge in the end came very quickly, they were talking about further away and then all of a sudden, yeah you can go” (PWS02).

Even for those who did feel involved in the discharge planning process, they experienced a lack of practical preparation. The lack of partnerships in goal-setting processes left PWS and CGs frustrated and with a disjointed understanding of their post-discharge trajectory.

“Again, it goes back to the goal setting. I would have loved in hospital to know when I would be able to go home, what it would look like. Like in the early days obviously I could do very little for myself, I would have loved to be told as you progress, once you can get around with the frame, if your home is suitable or whatever… Like I had been going home at weekends and then I had been home overnight at weekends, at that point I thought I can go home now because what is the difference with me being at home overnight and me being at home overnight for the week?” (PWS02).

PWS and CGs highlighted the challenges at transition to home when there was a breakdown in communication across HCPs and settings. The impact of a lack of coordination of care extended beyond discharge as neither GPs nor support staff were provided with relevant information e.g. regarding prescriptions and follow-up care that was required, hindering their ability to support rehabilitation and recovery. Expectations of “continuing everything” following discharge from rehabilitation wasn’t met as onward referrals to services weren’t made;

“Yeah, I thought it was referred. When they say you continue everything, I thought it meant everything I was doing in (rehab hospital) but it didn’t” (PWS06).

and mismatched timings of referrals meant that available supports didn’t align with the immediate needs of PWS

“they called us about two weeks ago but they were under the impression (X) was ready to go back to work so they wanted to work with him. So, she said when we are three months from going back to work, that is when we start working with people. I said, I can’t tell if (X) is three months away from working at the moment, I wouldn’t think so, I think probably wait another three or six months and then see where we are at” (CG06).

PWS expressed dissatisfaction with generic community services post-discharge, finding them inadequate for their needs. Similarly, CGs felt that the lack of specialism in community services impacted negatively on PWS to optimise their recovery. They described how professionals with stroke expertise could offer specialised input and directives for rehabilitation. For those who accessed more specialised community services, they emphasised the sense of encouragement for progress experienced in the more specialist environment

“it is very much you do an exercise, you pause, we try it again. It is a very much keep going, keep going, keep going, in hospital it was: oh no you might fall, you might trip……Yeah, facility wise they have far better facilities than the hospital. I know that their primary role is to do rehabilitation so maybe that would explain it but I found the hospital, and maybe it is because they were so busy, their aim was just to get through the day and one day just merged into the other” (PWS02).

PWS and CGs recognised that their residual needs over time extended beyond medical aspects of stroke and expressed a preference for a collaborative approach in determining appropriate services and support systems tailored to their needs.

“and the practical access to a team rather than necessarily waiting on the consultant or time with the consultant because the consultant is focused on the clinical side and that is their job, it is not necessarily holistic. So, I am talking about holistic access because your needs can vary dramatically. So, put simply, it is access to a team that can say, do you know what, here is a suggestion” (PWS05).

Feelings of injustice, disappointment and upset were expressed by participants as they waited for rehabilitation services and other supports to commence, and then for assessments to be repeated, which prolonged progression to next steps

“That is a disgrace because now we are being assessed again, we have to sit around, do nothing for about two months until they have all done their assessments. He has deteriorated and then they decide to work with him. It is just really annoying to be honest” (CG04).

Inconsistent social care experienced by stakeholders further complicated the waiting, causing delays in discharge, and feelings of uncertainty and helplessness.

“I was going to be let home based on a care plan being in place but I don’t think the care plan was ready at the end of November ….So I had I think it was Sally was her name, I had her for two days, then Rose came back from holidays, and between the two of them, either one or the other, then there was a whole week at Christmas that I had no one….Well Ger is the fifth person I have had up until the middle of January” (PWS06).

PWS and CGS who met a ‘coordinator’ within ESD or specialised community rehabilitation teams felt supported through their transition to home; whereas those who didn’t experience this type of support discussed a gap in care coordination. Central to participant preferences was a specific person to coordinate the journey: a versatile, accessible point of contact, offering guidance, bridging services, ensuring personalised care, and fostering collaboration for the benefit of PWS and CGs,

“it just needs to be a person with a brain who can coordinate things. So I think that link person is missing completely” (CG04).

and ensure continuity

“But if you had the case worker you would have the public health nurse, I guarantee you she would come in because you would have a case worker who knew what you needed” (PWS06).

IV. Inequity of access.

The experiences of PWS and CGs voiced in this theme highlights the reality of the disparity in access to resources and care during transition home after stroke. Rather than clearly defined preferences, it could be assumed participants would lean towards advocating for enhanced transparency in procedures and ensuring fair access.

Individuals talked about feeling discriminated against, experiencing an inequity in access to appropriate stroke recovery pathways, with those over 65 having comparatively smoother access to rehabilitation, despite sharing the same needs

“I said you are discriminating against people under 65. Because anyone over 65 would go to (the rehab unit). And she said that is just because it is elderly care. And I said, no, they are the same diagnosis, they are both stroke so you should not be picking out people who are over 65 to give them preferential treatment over under 65” (CG04).

While initiatives like ESD and specialised community services provided solace to some, regional disparities further exacerbated these inequalities, magnifying the stark divide between those with access and those without. Unequal access to crucial support was often tied to financial ability. Despite their medical needs, many PWS were ineligible for a medical card based on income criteria which impacted their access to crucial equipment and assistance to support discharge. Some were acutely aware that their opportunities for ongoing recovery differed from many others in a similar situation, and this was based on their ability to afford out-of-pocket expenses for essential supports

“maybe not everybody would be going for a private physio, like I am going three times a week for physio and paying for it. There might be lots of people who would love to do that but can’t do it, you know” (PWS01).

Many participants were frustrated by a lack of transparent processes to access financial support. This, alongside ineligibility for services due to income or financial barriers, was perceived by some as refusal of access to healthcare. One dyad recalled being advised to lobby politicians to access a medical card and an inpatient rehabilitation bed, and expressed their surprise coupled with a hint of expectation regarding the political aspect

“Actually, that is what really surprised me, well it does and it doesn’t surprise me, is how political it all is, the patient journey is incredibly political” (CG03).

## Discussion

This study explored the experiences and preferences for PWS and CGs in relation to supports at transition to home. Four key themes emerged, namely a (i) need for tailored information-sharing, at the right time, and in the right setting; (ii) the importance of emotional support; (iii) left in limbo; and (iv) inequity of access, reaffirming existing knowledge about the challenges in transitioning to home post-stroke, while providing fresh perspectives into preferences for supports at this juncture, aiding our understanding of the components necessary for effective intervention design.

The international literature echoes the frustration PWS and CGs with the lack of comprehensive information and inconsistent sharing, and highlights their desire for adequate information provision, especially about their condition and the healthcare system [35, 36]. “Being in the picture versus being in the dark” is a key enabler for community reintegration after stroke, suggesting that individuals empowered by clear information can re-engage in activities [37]. Globally, access to personal health information is recognised as a fundamental right [38], as it fosters control and confidence in understanding one’s own situation [39]. Similarly, this study found positive experiences among participants who actively sought or had access to their own health information. Receiving information specifically tailored to the diagnosis and needs of PWS, such as what to expect on discharge; availability of resources including support groups; the various post-stroke fatigue and psychological changes and support available; and practical information and support around return to driving and work, is considered ‘critical’ in increasing discharge readiness and reducing uncertainty through the transition period [8, 37, 40, 41]. PWS in this study described similar needs and preferences for individualised information. Receiving tailored information was associated with positive emotional wellbeing, emphasising the correlation between supportive communication channels and networks and emotional well-being. However, the lack of emotional support across the transition was a challenge, leading to a preference for more. Similarly, psychological support for PWS and their families is lacking internationally [41, 42]. Individuals in this study, and in the wider literature [41, 43], value peer support and “someone to talk to” to help them cope with emotional changes after stroke. Indeed, international guidelines [44] and stroke advocacy groups [45] recommend PWS and CGs be provided with information about peer support groups available in their community prior to discharge. Participants in this study suggest that earlier integration with stroke support organisations, even within the hospital setting, would be beneficial for PWS and their families.

Consistent with the research [5, 41, 46], our findings portray fragmented care coordination during stroke transition-to-home, with PWS and CGs ‘left in limbo’, feeling uninvolved in care decisions and treatment, and unprepared for discharge. Stroke-related information and residual needs should be relayed effectively, and be fully understood, in order to achieve a smooth coordinated transition from hospital to home [47]. However, participants in this study, and internationally [36, 40, 46], experience many communication gaps across service providers and settings. Active roles at both sender and receiver levels [47] are required; however, achieving effective communication is a challenging endeavour, often unattained [36, 47, 48]. The sense of waiting or being ‘left in limbo’ is echoed across the literature. In the United States very low level of PWS actually received home health services or outpatient rehabilitation at transition to home, and many faced long waits for essential services and supports [49]. This is further complicated in Ireland by the wide variability of post-discharge programme types and stroke expertise available for PWS, a fact that is highlighted in the literature [22], and again by PWS and CGs in this study. The lack of discharge planning, active PWS and CG involvement, timely and specialist post-discharge support, and multidisciplinary follow-up, are acknowledged as significant factors in the ability of PWS and CGs to cope with post-stroke changes, both in this study and in the literature [36]. Regions with established post-acute audits such as the Sentinel Stroke National Audit Programme (SNNAP) in the UK [50], allow for these challenges to be monitored and addressed; however, globally and in Ireland there are shortcomings in services capturing information beyond the hospital discharge [21, 23, 51]. The implementation of a transition framework, and the auditing of services beyond acute care, are recommended to ensure comprehensive care continuity and to address gaps in services that may occur after the acute phase of stroke treatment [6, 7, 48]. In terms of improving care coordination at transition, some individuals in this study benefited when members of ESD or specialised community services acted as coordinators liaising between the hospital and community services on their behalf; conversely those without such support felt a gap in this aspect of coordination. This led to study participants proposing a dedicated coordinator person be part of the transition process. Evidence around the effectiveness of this role is uncertain [17, 46, 52]. In the early ESD literature this coordinator role appears embedded within the team [53], while a community-based “transition specialist”, acting as a bridge across settings, sharing information, assisting patient engagement, and facilitating access to resources for a smoother transition has been recommended in a position paper on post stroke transition planning [48].

This study also highlights disparities in care experiences during the transition period including access to inpatient rehabilitation, and inequities influenced by financial ability, geographical location and political drive. Following acute stroke care in Ireland, the balance between public and private healthcare shifts to discussions about access, choice, and additional services for those willing or able to pay for them. The issues identified in this study are closely tied to the structural and resource-related factors of the Irish health system. However, similar challenges are also observed internationally. Historically, PWS of a lower socioeconomic status were less likely to receive evidence-based stroke services and more likely to experience severe post-stroke deficits due to increased barriers to accessing essential therapies and medications for optimal recovery. Stroke impacts, as measured by disability-adjusted life-years lost and mortality rates are over three times higher in low-income countries compared to high- and middle-income countries [54]. Inequitable access to stroke unit care was also evident during the early years, but diminished internationally and in Ireland as the total capacity for stroke unit care increased [42, 55]. However, as is the case in this study, inequity in access to rehabilitation persists, regardless of socioeconomic status, with variable availability internationally to inpatient rehabilitation, early supported discharge programmes, and community based rehabilitation [56]. Eligibility criteria for rehabilitation access are not uniform, evidence determining who will benefit most is scarce, and a dearth of bed availability are common barriers [57]. The regional disparity identified in this study, showing that more rural participants with moderate to high needs experienced longer hospital stays, with one being discharged directly home without ESD or inpatient rehabilitation, likely highlights the challenges of accessing rehabilitation beds and community support in rural areas. This finding is consistent with the international literature, where people in non-urban hospitals often have inferior access to best-practice stroke care and key stroke interventions [58], presumed attributable to smaller populations dispersed over wider geographical areas. PWS and CGs in this study recommend increased transparency in service and support availability, with equitable access for all those transitioning to home. This study recommends extending the scope of the national stroke audit [42] beyond acute services to track the adoption of evidence-based stroke guidelines [7], in order to monitor and help address inequities in access and quality of stroke care. Tracking access to rehabilitation would also contribute towards the Rehabilitation 2030 initiative [59], which aims to scale up and strengthen rehabilitation services worldwide, and enhance the quality of life for people with disabilities.

### Strengths and limitations

This qualitative study elicited rich information from the perspectives of PWS and CGs on the supports they received or would like to receive at transition to home. The identified themes describe key components essential for improving the transition to home after stroke which will greatly inform future service development aimed at enhancing the transition process. This study adhered to COREQ reporting guidelines [19], and addressed methodological rigor by considering 20 critical questions to guide quality assessment of RTA research [33]. Incorporating PPI perspectives enriched all aspects of this study, from informing study design to contributing to interpretation of the findings and recommendations for policy and practice. This collaborative approach ensured that the study’s discussion was grounded in the lived experiences and insights of those most directly affected by stroke. By integrating researcher reflexivity around data-analysis and data interpretation, with PPI collaboration, the quality, validity and impact of the research was enhanced. The findings of this study should be interpreted in the context of a number of limitations. By not capturing transition support experiences and preferences as they evolved over time we may have limited the generalisability of these findings. The timing of the interviews, which were at a single time-point, and heterogeneous in nature (range 1–6 months post-discharge) might also be considered a limitation, as variations in discharge times may have influenced the support experiences and preferences of those with stroke. However, this heterogeneity likely reflects real-world post-discharge scenarios thus enhancing the ecological validity of the study. The study’s small sample size may not have identified other significant themes, especially those relevant to specific sub-populations. Further research with larger and more diverse samples is needed to uncover additional themes and understand their relative importance across different sub-populations in order to enhance the comprehensiveness and applicability of the study findings. We acknowledge that the perspectives of healthcare professionals who deliver support during transition to home is crucial, yet absent in this publication. We also acknowledge that caregivers recruited were spouses or family members of PWS, and did not include the perspectives of formal caregivers, which may result in a less comprehensive, and a missed opportunity, for greater understanding in the area. Future research should address both the perspectives of healthcare professionals and formal caregivers.

## Conclusion

This study emphasises that regardless of the discharge route, and despite formal support systems in place, PWS and families in Ireland encounter challenges and care fragmentation during the transition period. The transition support experiences and the preferences of PWS and CGs during this critical period highlight the need for better communication and care coordination; early and ongoing access to emotional support; and equitable access to tailored services and support. Furthermore, to enhance a patient-centred transition, preferences for support include adopting a collaborative partnership approach that fosters enhanced collaboration among PWS, CGs, healthcare professionals and support organisations. This includes actively involving PWS and their CGs in collaborative goal-setting and decisions regarding care. By outlining the key elements of the Irish health system, this study underscores the importance of considering local healthcare contexts when addressing the needs of PWS. Findings will contribute to a co-design process focussed on the essential components of an intervention to support the transition to home after stroke within the Irish context.

### Electronic supplementary material

Below is the link to the electronic supplementary material.

Supplementary Material 1

## Data Availability

The datasets used and/or analysed during the current study are available from the corresponding author upon reasonable request (gocallaghan@rcsi.com).
