# Supplementary material for: Experiences and preferences of people with stroke and caregivers, around supports provided at the transition from hospital to home: a qualitative descriptive study
Source: BMC Neurol. 2024 Jul 22;24:251. doi: 10.1186/s12883-024-03767-0 (PMC11265157; doi:10.1186/s12883-024-03767-0)
Supplement: Supplementary file 1 — Supplementary Material 1 [file 12883_2024_3767_MOESM1_ESM.docx]

**Supplemental Data:**

**Preferences and Experiences of People with Stroke and Caregivers, around Supports Provided at the Transition from Structured Stroke Care to Home: A qualitative descriptive study**

**Authors**

Geraldine O’Callaghan, Martin Fahy, Sigrid O’Meara, Sebastian Lindblom, Lena von Koch, Peter Langhorne, Rose Galvin, Frances Horgan

**Contents**

**S1.** Consolidated criteria for reporting qualitative studies (COREQ): 32-item checklist 2

**S2.** Interview Guide (Sample provided for PWS and CG) 5

**S3.** Table I: Maximum Variation Sampling to recruitment of PWS, CG and HCP 14

**S1. Consolidated criteria for reporting qualitative studies (COREQ): 32-item checklist**

| **No. Item** | **Guide questions/description** | **Reported on Page #** |
| --- | --- | --- |
| **Domain 1: Research team and reﬂexivity** |  |  |
| ***Personal Characteristics*** |  |  |
| 1. Inter viewer/facilitator | Which author/s conducted the interview or focus group? | 6 |
| 2. Credentials | What were the researcher’s credentials? E.g. PhD, MD | 6 |
| 3. Occupation | What was their occupation at the time of the study? | 6 |
| 4. Gender | Was the researcher male or female? | 6 |
| 5. Experience and training | What experience or training did the researcher have? | 6 |
| ***Relationship with participants*** |  |  |
| 6. Relationship established | Was a relationship established prior to study commencement? | 4 |
| 7. Participant knowledge of the interviewer | What did the participants know about the researcher? e.g. personal goals, reasons for doing the research | 5 |
| 8. Interviewer characteristics | What characteristics were reported about the inter viewer/facilitator? e.g. Bias, assumptions, reasons and interests in the research topic | No |

| **Domain 2: study design** |  |  |
| --- | --- | --- |
| ***Theoretical framework*** |  |  |
| 9. Methodological orientation and Theory | What methodological orientation was stated to underpin the study? e.g. grounded theory, discourse analysis, ethnography, phenomenology, content analysis | 6 |
| ***Participant selection*** |  |  |
| 10. Sampling | How were participants selected? e.g. purposive, convenience, consecutive, snowball | 4 |
| 11. Method of approach | How were participants approached? e.g. face-to-face, telephone, mail, email | 4/5 |
| 12. Sample size | How many participants were in the study? | 5 |
| 13. Non-participation | How many people refused to participate or dropped out? Reasons? | N/A |
| ***Setting*** |  |  |
| 14. Setting of data collection | Where was the data collected? e.g. home, clinic, workplace | 5 |
| 15. Presence of non-participants | Was anyone else present besides the participants and researchers? | 5 |
| 16. Description of sample | What are the important characteristics of the sample? e.g. demographic data, date | 7/8 |
| ***Data collection*** |  |  |
| 17. Interview guide | Were questions, prompts, guides provided by the authors? Was it pilot tested? | 7 and Supplemental data S2 |
| 18. Repeat interviews | Were repeat inter views carried out? If yes, how many? | N/A |
| 19. Audio/visual recording | Did the research use audio or visual recording to collect the data? | 2 |
| 20. Field notes | Were ﬁeld notes made during and/or after the interview or focus group? | 7 |
| 21. Duration | What was the duration of the inter views or focus group? | 6 |
| 22. Data saturation | Was data saturation discussed? | 5 |
| 23. Transcripts returned | Were transcripts returned to participants for comment and/or correction? | 6 |
| **Domain 3: analysis and ﬁndings** |  |  |
| *Data analysis* |  |  |
| 24. Number of data coders | How many data coders coded the data? | 6 |
| 25. Description of the coding tree | Did authors provide a description of the coding tree? | We initially included a coding tree visual. However, upon review, we found that it did not enhance the manuscript's clarity or depth, and have subsequently omitted it. |
| 26. Derivation of themes | Were themes identiﬁed in advance or derived from the data? | 6 |
| 27. Software | What software, if applicable, was used to manage the data? | 6 |
| 28. Participant checking | Did participants provide feedback on the ﬁndings? | No |
| ***Reporting*** |  |  |
| 29. Quotations presented | Were participant quotations presented to illustrate the themes/ﬁndings? Was each quotation identiﬁed? e.g. participant number | 8-14 |
| 30. Data and ﬁndings consistent | Was there consistency between the data presented and the ﬁndings? | 8-14 |
| 31. Clarity of major themes | Were major themes clearly presented in the ﬁndings? | 8-14 |
| 32. Clarity of minor themes | Is there a description of diverse cases or discussion of minor themes? | N/A |

**S2. Interview guide**

Note: The interview guides adhered to a consistent theme, with modifications made to accommodate the unique perspectives of each stakeholder type and the specific discharge pathways**.**

**STUDY**

**Preferences and Experiences of People with Stroke and Caregivers, around Supports Provided at the Transition from Structured Stroke Care to Home: A qualitative descriptive study**

**Stakeholder**: Stroke Survivors

**Discharge Pathway:** Acute/Rehab to home

**Introduction to interview**

How are you today? Thank you so much for agreeing to take part in this study, I'm really, grateful to you and for your time.

**Background:**

Stroke is one of the leading causes of death and disability worldwide. In Ireland over 5,500 people are hospitalised with a stroke every year, with nearly 60% of all stroke patients discharged home. The process of transitioning from hospital to home and back into the community, taking into account their unique and complex needs, can be challenging for people who have experienced a stroke and their families. The aim of this study is to explore hospital / early supported discharge (ESD) to home transition experiences, and preferences for supports to be provided at this juncture. This information will inform the development of an intervention to improve transitions home after stroke.

I would like to start by asking you some questions about your stroke, and specifically how you experienced the transition to home. From the interview, I hope to understand more about your experience of transitioning home after stroke, and your views on supports for stroke survivors and their families at the transition to home.

- I will be recording the interview so that I have an accurate record of what you tell me. The interview is confidential, and you will not be identifiable when it is typed up.
- I will also send you a copy of the interview so that you can check that it accurately reflects what was said and you are free to make any changes.
- There are no right or wrong answers in this interview, there is just your **experiences**. If there are any questions that you don't understand, please stop me and ask me to explain or rephrase them.
- The interview should last about 30-40 minutes, but if you would like to take a break before that, please just let me know. If we go on a bit longer than that and you are feeling tired, please let me know and we will pause or stop.

**Do you have any questions for me about the study or the plan for the interview before we start?**

Now I am going to turn on the recorder. I am going to press record, and record the date and start time of our interview and then we will begin the interview.

**Interview Prompts Stroke survivors Acute to home Rehab to home**

(Pre-discharge and at discharge directly home from hospital)

**1a. Firstly we are going to talk about your experience in the days leading up to coming home, and the day you were discharged**.

- When you were in hospital what information were you given about stroke? (Prompt: education about stroke and stroke diagnosis; recovery and rehabilitation)
  - - Who delivered that information?, How did you receive this information?, Where was it delivered?, When did you receive this information?
    - Did you feel ready to receive this information?
- When you were in hospital what information were you given to support life after stroke? (Prompt: medicine; nutrition; exercise; financial support and accessing entitlements; return to driving/accessing blue badge; return to work)
  - - Who delivered that information?, How did you receive this information?, Where was it delivered?, When did you receive this information?
    - Did you feel ready to receive this information?
- In the lead up to you being discharged from hospital who talked to you about going home?
- What services/information were offered to you to prepare you for going home? (Prompt: H/V to assess environment; Case conference)
- Who offered you this support?, How was this support provided?, Where was it provided?
- What details were you given about how your continuous rehabilitation needs would be met?
  - What specific info were you given about the community team (Prompt: to whom referrals were made, whom the team comprised, how to contact them, where it would be provided),
  - Who provided the information?, How did you receive this information?, When did you receive this information?, Where was it delivered?
  - How did you feel about going home?
- How was your family involved in preparation for discharge with ESD ( Prompt: family meeting to discuss your needs and their capacity; carer training)
  - - Who offered your family support?, How was the support provided to your family?, Where did your family receive this support?
- Were any other services offered to/ set up for you, in hospital, that could support you after discharge (Prompt: Home support; ABI; IHF programmes)
- What do you remember about the day you were discharged home from the hospital? (Prompt: 3 key things – what you experienced / how you felt)
- On a scale of 0-10, (0 being unprepared, 10 being very prepared), how prepared did you feel going home?

10

0

- - What would have needed to happen in the days leading up to, and at discharge, to increase your feeling of being prepared to go home?
- How would you describe the transition to home experience?
- How involved did you feel in your own discharge process?
  - How important is it for you to be involved in planning your discharge and care after stroke? Why?

**2. I want you to go back to the days leading up to your discharge….close your eyes if you like…. Imagine if everything in the discharge planning was perfect….what would a perfect discharge look like for you?**

(Prompt: What discharge supports/ information would you like to receive? Describe the support.)

- Who should provide this information / support?, How would it be provided?, Where would it be provided?, At what point in your hospital / pre-discharge care would it be delivered?

(Post-discharge from hospital)

**1b. We will now consider what it was like in the days and weeks after you came home from hospital.**

- What ongoing rehabilitation / support did you receive in the first few days and weeks when you got home from hospital?
- Who came to see you / did you go somewhere to access ongoing rehabilitation or support?
- Exactly what support did they provide?
  - Did you receive information or support around other needs such as emotional support to care for yourself, financial support and accessing entitlements; return to driving/accessing blue badge; return to work? - Who provided it; How was it provided? Where was it provided?
  - Were you signposted to community programmes (IHF, stroke support groups, community exercise groups)? – Who provided it; How was it provided?
  - What advice or support do you need, then or now, that you haven’t yet received? (Prompt: ongoing rehabilitation, signposting to a support group, information on activities such as driving/accessing blue badge, information on accessing entitlements and financial support; advice on return to work; advice on managing fatigue…..anything else)

**2. How did you feel when you were discharged from hospital, what would you like to see done differently?**

**In an ideal world, what would the perfect discharge look like? if resources were unlimited / you had a magic wand what would ”perfect support” in the days after discharge, and in the first 4-6 weeks home look like for you? Describe the ‘support’**.

- Who would provide this support?, How would it be provided? , Where would it be provided?
- At what point in your post-discharge care?
- For how long?
- **What do you consider is the role of in suporting people with stroke in the community.**
- **GP**
- **PHN**
- **Psychologist**
- **SW**
- **Dietican**
- **PT, OT, SLT**

W

S

**3. Let’s consider your experience of coming home after stroke in the context of**

**the health services**

- What do the health service do well?
- What do they not do well?
- How could health service improve more?

O

- In your opinion what is limiting the health service from providing

T

the best possible discharge support and experience to stroke

survivors and their families

**End of Interview:**

Thank you so much for talking to me today, I've asked all of my questions now. **Is there anything that you would like to say that we did not discuss or something that you haven't had the opportunity to say?**

**Post interview – recording off:**

We are at the end of the interview, I'm going to turn off the recorder now and will record the time again. Now that that the interview is completed, how do you feel, were you happy with everything?

I want to say a sincere thank you for taking part in the study. I'm enormously grateful for your honesty and openness today. Remembering and talking about this difficult time may not have been easy for you, so thank you for sharing with me.

**So what will happen next**? I will transcribe/ type up the audio recording of our interview; I will remove any personal details; and then I will spend some time analysing your interview data with data from other participants.

Would you like to receive a copy to check the details? How would you like to receive the transcript?

In the meantime, if you have any questions or queries, feel free to contact me.

**Geraldine O’Callaghan. Email:**gocallaghan@rcsi,com **Telephone:** 085-8443599

It was a pleasure to talk to you, thank you again, and enjoy the rest of your day.

**Study**

**Experiences and preferences of stroke survivors’, carers’ and healthcare professionals’ for supports provided at transitions from structured stroke services to home.**

**Stakeholder**: Caregivers

**Discharge Pathway**: Acute/Rehab to home

**Introduction to interview**

How are you today? Thank you so much for agreeing to take part in this study, I'm really grateful to you and for your time.

**Background:**

Stroke is one of the leading causes of death and disability worldwide. In Ireland over 5,500 people are hospitalised with a stroke every year, with nearly 60% of all stroke patients discharged home. The process of transitioning from hospital to home and back into the community, taking into account their unique and complex needs, can be challenging for people who have experienced a stroke and their families. The aim of this study is to explore hospital / early supported discharge (ESD) to home transition experiences, and preferences for supports to be provided at this juncture. This information will inform the development of an intervention to improve transitions home after stroke.

I would like to start by asking you some questions about your loved ones stroke, and specifically how you experienced his/her transition to home. From the interview, I hope to understand more about your experience of your loved one transitioning home after stroke, and your views on supports for stroke survivors and their families at the transition to home.

• I will be recording the interview so that I have an accurate record of what you tell me. The interview is confidential, and you will not be identifiable when it is typed up.

• I will also send you a copy of the interview so that you can check that it accurately reflects what was said and you are free to make any changes.

• There are no right or wrong answers in this interview, there is just your experiences. If there are any questions that you don't understand, please stop me and ask me to explain or rephrase them.

• The interview should last about 30-40 minutes, but if you would like to take a break before that, please just let me know. If we go on a bit longer than that and you are feeling tired, please let me know and we will pause or stop.

Do you have any questions for me about the study or the plan for the interview before we start?

Now I am going to turn on the recorder. I am going to press record, and record the date and start time of our interview and then we will begin the interview.

**(Pre-discharge and at discharge directly home from hospital)**

1a. Firstly we are going to talk about your experience in the days leading up to your loved one coming home, and the day he/she was discharged.

• What information were you given about your loved ones stroke? (Prompt: education about stroke and stroke diagnosis; recovery and rehabilitation)

 Who delivered that information?, How did you receive this information?, Where was it delivered?, When did you receive this information?

 Did you feel ready to receive this information?

• In the lead up to your loved one being discharged from hospital who talked to you about him/her coming home?

• What details were you given about your loved ones continuous rehabilitation needs and how these would be met?

o What specific info were you given about the community teams (Prompt: to whom referrals would be made, whom the community team comprised, how to contact them, where it would be provided),

o Who provided the information?, How did you receive this information?, When did you receive this information?, Where was it delivered?

• What services were offered to you to prepare you for your loved one coming home? (Prompt: H/V to assess environment; family meeting to discuss your needs and their capacity; carer training)

 Who offered you this support?, How was this support provided?, Where was it provided?

• What information was given to you to help support his/her life after stroke? (Prompt: education around medicine and nutrition; financial support and accessing entitlements; accessing blue badge; emotional support; advice on balancing work and caring role)

 Who delivered that information?, How did you receive this information?, Where was it delivered?, When did you receive this information?

 Did you feel ready to receive this information?

• Were any other services offered to/ set up for you, in hospital, that could support you after your loved ones discharge (Prompt: Home support; ABI; IHF programmes)

• What do you remember about the day your loved one was discharged home from the hospital? (Prompt: 3 key things – what you experienced / how you felt)

• On a scale of 0-10, (0 being unprepared, 10 being very prepared), how prepared did you feel for your loved one coming home?

o What would have needed to happen in the days leading up to, and at discharge, to increase your feeling of being prepared for your loved one to come home?

• How would you describe the transition to home experience?

• How involved did you feel in your loved ones discharge process?

o How important is it for you to be involved in planning your loved ones discharge and care after stroke? Why?

2. I want you to go back to the days leading up to your loved ones discharge….close your eyes if you like…. Imagine if everything in the discharge planning was perfect….what would a perfect discharge look like for you? Describe it.

(Prompt: What discharge supports/ information would you like to receive?

o Who should provide this information / support?, How would it be provided?, Where would it be provided?, At what point in your hospital / pre-discharge care would it be delivered?

**(Post-discharge from hospital)**

1b. We will now consider what it was like in the days and weeks after your loved one came home from hospital.

• How soon did ongoing rehabilitation start? Who visited your loved one in the first week?

• What support did you receive in the first few days and weeks when your loved one came home from hospital?

o Who came to see you and what support did they provide?

o Did you receive information or support around other needs such as emotional support to care for yourself, financial support and accessing entitlements; accessing blue badge; balancing work and a caring role? - Who provided it; How was it provided? Where was it provided?

o Were you signposted to community programmes (IHF, carer support groups)? – Who provided it; How was it provided?

o What advice or support do you need, then or now, that you haven’t yet received? (signposting to a support group, information on accessing blue badge, information on accessing entitlements and financial support; advice on balaning work and caring role; …..anything else)

2. From a caregivers perspective, what would the perfect discharge look like? if resources were unlimited / you had a magic wand what would ”perfect support” in the days after your loved ones discharge, and in the first 4-6 weeks home look like for you? Describe the ‘support’.

o Who would provide this support?, How would it be provided?, Where would it be provided?

o At what point in your loved ones post-discharge care?

o For how long?

• What do you consider is the role of in suporting people with stroke and their families in the community.

• GP

• PHN

• Psychologist

• SW

• Dietican

• PT, OT, SLT

3. Let’s consider your experience of your loved one coming home after stroke in the context of

the health services

• What do the health service do well?

• What do they not do well?

• How could health service improve more?

• In your opinion what is limiting the health service from providing

the best possible discharge support and experience to stroke

survivors and their families

**End of Interview:**

Thank you so much for talking to me today, I've asked all of my questions now. Is there anything that you would like to say that we did not discuss or something that you haven't had the opportunity to say?

**Post interview – recording off:**

We are at the end of the interview, I'm going to turn off the recorder now and will record the time again.Now that that the interview is completed, how do you feel, were you happy with everything?

I want to say a sincere thank you for taking part in the study. I'm enormously grateful for your honesty and openness today. Remembering and talking about this difficult time may not have been easy for you, so thank you for sharing with me.

So what will happen next? I will transcribe/ type up the audio recording of our interview; I will remove any personal details; and then I will spend some time analysing your interview data with data from other participants.

Would you like to receive a copy to check the details? How would you like to receive the transcript?

In the meantime, if you have any questions or queries, feel free to contact me.

Geraldine O’Callaghan. Email: gocallaghan@rcsi.com Telephone: 085-8443599

It was a pleasure to talk to you, thank you again, and enjoy the rest of your day.

**S3. Table I: Maximum Variation Sampling to recruitment of PWS, CG and HCP**

| **PWS*** | **Severity of need** | | **Total** | **CGs** | **Geographic Location** | | | **Total** |
| --- | --- | --- | --- | --- | --- | --- | --- | --- |
|  | **High** | **Low** |  |  | **Site 1** | **Site 2** | **Site 3** |  |
| Home direct | 2 | 1 | 3 | Supporting home direct | 1 | 1 | 1 | 3 |
| Home after inpatient rehabilitation | 1 | 1 | 2 | Supporting after inpatient rehabilitation | 1 |  | 1 | 2 |
| Home via ESD | 2 | 2 | 4 | Supporting  Home via ESD | 1 | 1 |  | 2 |
| **Total** | 5 | 4 | 9 | **Total** | 3 | 2 | 2 | 7 |
| *Unable to recruit a person with low need in site 1 | | | | | | | | |
